# Lysine Acetylation in the Proteome of Renal Tubular Epithelial Cells in Diabetic Nephropathy

**DOI:** 10.3389/fgene.2021.767135

**Published:** 2021-11-25

**Authors:** Jiayi Wan, Mingyang Hu, Ziming Jiang, Dongwei Liu, Shaokang Pan, Sijie Zhou, Zhangsuo Liu

**Affiliations:** ^1^ Department of Nephrology, the First Affiliated Hospital of Zhengzhou University, Zhengzhou, China; ^2^ Research Institute of Nephrology, Zhengzhou University, Zhengzhou, China; ^3^ Henan Province Research Center for Kidney Disease, Zhengzhou, China; ^4^ Key Laboratory of Precision Diagnosis and Treatment for Chronic Kidney Disease in Henan Province, Zhengzhou, China

**Keywords:** proteome, lysine acetylation, renal tubular epithelial cells, diabetic nephropathy, acetyltransferase

## Abstract

Diabetic nephropathy is considered one of the most common microvascular complications of diabetes and the pathophysiology involves multiple factors. Progressive diabetic nephropathy is believed to be related to the structure and function of the tubular epithelial cells in the kidney. However, the role of lysine acetylation in lesions of the renal tubular epithelial cells arising from hyperglycemia is poorly understood. Consequently, in this study, we cultured mouse renal tubular epithelial cells *in vitro* under high glucose conditions and analyzed the acetylation levels of proteins by liquid chromatography-high-resolution mass spectrometry. We identified 48 upregulated proteins and downregulated 86 proteins. In addition, we identified 113 sites with higher acetylation levels and 374 sites with lower acetylation levels. Subcellular localization analysis showed that the majority of the acetylated proteins were located in the mitochondria (43.17%), nucleus (28.57%) and cytoplasm (16.19%). Enrichment analysis indicated that these acetylated proteins are primarily associated with oxidative phosphorylation, the citrate cycle (TCA cycle), metabolic pathways and carbon metabolism. In addition, we used the MCODE plug-in and the cytoHubba plug-in in Cytoscape software to analyze the PPI network and displayed the first four most compact MOCDEs and the top 10 hub genes from the differentially expressed proteins between global and acetylated proteomes. Finally, we extracted 37 conserved motifs from 4915 acetylated peptides. Collectively, this comprehensive analysis of the proteome reveals novel insights into the role of lysine acetylation in tubular epithelial cells and may make a valuable contribution towards the identification of the pathological mechanisms of diabetic nephropathy.

## Introduction

Diabetic nephropathy (DN) is regarded as one of the most common microvascular complications of diabetes and the pathophysiology involves multiple factors. As one of the major pathological manifestations of diabetic nephropathy, renal tubulointerstitial fibrosis is strongly related to damage of the renal tubular epithelial cells arising from glucose, hypoxia, and inflammatory factors (Yang et al., 2020). It is now well established from a variety of studies that the inhibition of apoptosis, pyroptosis, autophagy, and metabolic abnormalities may protect the structure and function of the renal tubular epithelial cells cultured with high glucose (Qin et al., 2018; Lv et al., 2019; Zhu et al., 2020).

Protein acetylation is a post-translational modification (PTM) process in which acetyl groups are bound to amino acids by acetyltransferase. This modification, which is common in eukaryotes and prokaryotes, is vital to the regulation of enzyme activity, protein stability and signaling pathways (Kouzarides, 2000). Lysine acetylation, as a form of protein acetylation modification, is the most extensively investigated form of reversible protein modification. Previous studies have concentrated on lysine acetylation in histones and its effects on gene expression and chromatin remodeling (Shen et al., 2015). However, much of the research conducted over recent years have shown that non-histone protein acetylation is also involved in the regulation of the molecular biological function of cells (Narita et al., 2019).

Numerous experiments have shown that certain proteins will undergo acetylation modification when stimulated by high glucose. The influence of glucotoxicity on the acetylation level of intracellular proteins in key cells, including pancreatic β cells, hepatocytes, neural stem cells, and osteoclasts, is closely linked to cell function and even the occurrence and development of diseases (Zhang et al., 2019; Zhou et al., 2019; Elumalai et al., 2020; Guo et al., 2021). Furthermore, the acetylation of DNA/RNA binding transcription factor Yin Yang 1 (YY1) in renal tubular epithelial cells under high glucose conditions has been shown to increase epithelial-mesenchymal transition (EMT) (Du et al., 2021). Reductions in the acetylation of p53 may attenuate the EMT process (Mizuguchi et al., 2012). Furthermore, the overexpression of deacetylase Sirtuin3 (Sirt3) can also regulate mitochondrial oxidative stress and mitigate apoptosis in Human kidney 2 (HK-2) cells (Jiao et al., 2016).

Although protein acetylation modifications are known to be involved in the pathogenesis of diabetic kidney disease, there are little related studies on acetylated proteome (Chen et al., 2014; Mi et al., 2016; Wang et al., 2021). Consequently, in the present study, we determined protein content and acetylation levels in mouse proximal renal tubular epithelial cells cultured with high glucose *in vitro* by applying an immunoaffinity enrichment strategy and liquid chromatography-mass spectrometry (LC-MS/MS). We identified 4311 acetylation sites on 2012 acetylated proteins. We also identified a number of significantly enriched pathways and proteins by screening the Kyoto Protocol Encyclopedia of Genes and Genomes (KEGG) and STRING databases. To our knowledge, this is the first comprehensive analysis of acetylation modifications in renal tubular epithelial cells under conditions of high glucose. Taken together, this information not only significantly improved our understanding of protein acetylation modification in DN, but also greatly assisted us in finding new approaches for the prevention and treatment of DN.

## Materials and Methods

### Cell Culture

A mouse proximal renal tubular epithelial cell line (TKPT) was purchased from ATCC^1^ and cultured in T75 cell culture vials. Cells were cultured in Dulbecco’s Modified Eagle’s Medium (DMEM) (Thermo Fisher Scientific), 10% fetal bovine serum (Thermo Fisher Scientific), 1% penicillin/streptomycin solution (Thermo Fisher Scientific) and glucose. Cells were either cultured with normal glucose levels (5.6 mM glucose) or high glucose levels (40 mM glucose) in an incubator at 37°C and 5% CO_2_ for 48 h.

### Western Blotting Analysis

Proteins were extracted from TKPT cells by radio immunoprecipitation assay (RIPA) lysis buffer (Beijing Soleibao Technology Co., Ltd) and protein concentrations were detected with a bicinchoninic acid (BCA) protein assay kit (Beijing Soleibao Technology Co., Ltd). Fifteen micrograms of each protein sample were then loaded into individual wells on a 12% gel separated by sodium dodecyl sulfate-polyacrylamide gel electrophoresis (SDS-PAGE) and transferred into a polyvinylidene fluoride (PVDF) membrane at 250 mA for 90 min. The blockage was performed with 5% skim milk/Tris-buffered saline with 0.1% Tween ® 20 detergent (TBST). The membranes were then washed with TBST three times for 5 min and incubated with anti-acetyl-lysine antibody (PTM Biolabs, Hangzhou, China) overnight at 4°C. The following day, after cleaning the membrane three times with TBST, the membrane was incubated with the secondary antibody at ambient temperature for 2 h. Lastly, the electrochemical luminescence (ECL) (Dalian Meilun Biotechnology Co., Ltd) was used to visualize the protein bands on the AI600 machine (Thermo Fisher Scientific).

### Proteomics Procedures

#### Sample Preparation and Protein Extraction

TKPT cells were cultured in T75 cell culture vials with DMEM (Thermo Fisher Scientific), 10% fetal bovine serum (Thermo Fisher Scientific), 1% penicillin/streptomycin solution (Thermo Fisher Scientific) and glucose at 37°C and 5% CO2 for 48 h. Three replicates were set for the control group (5.6 mM glucose) and the experimental group (40 mM glucose). The density of cells was 1 × 10^8^ in each group. Afterwards, the cells were whashed for three times with pre-cooled PBS and collected into 1.5-ml microcentrifuge tubes with a cell scraper. Samples were stored at −80°C to await analysis. Next, four volumes of cracking buffer (8 M urea, 1% protease inhibitor, 3 μM Trichostatin A, 50 mM Nicotinamide) were added to each sample and ultrasonic cracking was performed. Then, the samples were centrifuged at 12000 × g at 4°C for 10 min to remove cell debris. The supernatant was transferred to a new centrifuge tube and protein concentrations were determined with a BCA kit.

#### Trypsin Digestion and Tandem Mass Tag Labeling

For each sample, 0.7 mg of protein material per microcentrifuge tube was reduced by 5 mM dithiothreitol (DTT) for 30 min at 56°C. Iodineacetamide (IAA) was then added to a final concentration of 11 mM and incubated at ambient temperature in darkness for 15 min. Then, 20% trichloroacetic acid (TCA) was added and proteins were precipitated at 4°C for 2 h. Finally, the samples were centrifuged at 4500 × g for 5 min. The supernatant was then discarded, and the pellet was washed 2–3 times with pre-cooled acetone for protein precipitation The precipitate was allowed to air-dry and triethylammonium bicarbonate (TEAB) was added to a final concentration of 200 mM. After ultrasonic dispersion and precipitation, trypsin was added to a protease: protein ratio of 1:50 and enzymatic hydrolysis was carried out overnight.

Following hydrolysis with trypsin, peptides were dehydrated with Strata X C18 (Phenomenex) and freeze-dried in a vacuum. The peptides were then dissolved with 0.5 M TEAB and labeled with a tandem mass tag (TMT) kit in accordance with the manufacturer’s instructions. The simple operations were as follows: the labeling reagent was dissolved with acetonitrile and mixed with the peptides resuspended by TEAB. After the mixture was incubated at room temperature for 2 h, 5% hydroxylamine was added to terminate the reaction. Samples with different labels were mixed together and vacuum freeze-dried after desalting with Strata X C18 (Phenomenex).

#### HPLC Fractionation, Affinity Enrichment and LC-MS/MS Analysis

Samples were fractionated by high pH reverse-phase HPLC using Agilent 300Extend C18 column (5 μm particles, 4.6 mm ID, 250 mm length). Briefly, peptides were first separated using a gradient of 8–32% acetonitrile in 10 mM NH4CO3 (pH 9) over 60 min into 60 fractions. The injection volume was 20 μL, and the same sample was repeated 6 times. Then, the peptides were combined into nine fractions and dried by vacuum centrifuging.

For acetylated peptides enrichment, 3 mg trypsinized peptides dissolved in NETN buffer (100 mM NaCl, 1 mM EDTA, 50 mM Tris–HCl, 0.5% NP-40, pH 8.0) were incubated with 20 μL agarose beads coupled to anti-acetyl-lysine antibody PTM-104 (PTM Biolabs, Hangzhou, China) at 4°C overnight with gentle shaking. The beads were washed four times with NETN buffer and twice with ddH2O. The bound peptides were eluted from the beads with 0.1% trifluoroacetic acid. The eluted fractions were combined and vacuum-dried. The obtained peptides were cleaned with C18 ZipTips (Millipore) according to manufacturer instructions.

Next, the peptides were separated by an EASY-NLC 1200 ultra-performance liquid system as follows: mobile phase A was an aqueous solution containing 0.1% formic acid and 2% acetonitrile; mobile phase B consisted of the aqueous solution containing 0.1% formic acid and 90% acetonitrile (liquid gradient setting: 0–40 min, 6%–22% B; 40–54 min, 22%–32% B; 54–57 min, 32%–80% B; 57–60 min, 80% B; flow rate was maintained at 500 nL/min. Following separation, the peptides were injected into an NSI ion source and analyzed using Q Exactive™ HF-X mass spectrometry. The ion source voltage was set to 2.1 kV; the parent ion and secondary fragment of the peptide were detected and analyzed with a high-resolution Orbitrap. For MS scans, the m/z scan range was 350–1,600 and the scan resolution was set to 120,000; the resolution for HCD spectra was set to 30,000 at m/z 100. Data were collected using data-dependent acquisition (DDA) program; following the primary scan, the top 20 peptide parent ions with the highest signal intensities were selected and successively entered into an HCD collision pool for fragmentation with 28% collision energy, and secondary mass spectrometry was conducted. To improve the efficiency of mass spectrometry, we used the following parameter settings: automatic gain control (AGC) was set to 1E5; the signal threshold was set to 8.3E4 ions/s; the maximum injection time was set to 60 ms, and the dynamic exclusion time for tandem mass spectrometry was set to 30 s to avoid repeated exploration of the parent ions.

#### Database Searches

The MS/MS data obtained were processed using MaxQuant with an integrated Andromeda search engine (v.1.6.15.0). First, the database was set to Mus_musculus_10090 (20395 sequences); then an inverted database was used to calculate false positive rates (FDR) due to random matches. We also included a common database for contamination to remove the influence of contaminated proteins in the identification results. Second, the method of enzymatic digestion was set to Trypsin/P; the number of missing bits was set to 2; the minimum length of the peptide was set to 7 residues, and the maximum number of peptide modifications was set to 5. Third, the tolerance for mass errors with regards to the primary parent ions in the first search and the main search was set to 20 parts per million (PPM) and 4.5 PPM, respectively. The tolerance for mass error with regards to the secondary fragment ions was set to 20 PPM. Fourth, carbamidomethylation on Cys was specified as a fixed modification; oxidized methionine, acetylated protein N-termini, deamidation (NQ) and acetylation on Lys were set as variable modifications. Finally, the quantitative method was set as TMT-6plex, and the FDR for protein identification and PSM identification was set to 1%. Protein identifications were based on a minimum of one unique peptides.

### Bioinformatics Analysis

#### Functional Annotation of Proteins and Enrichment Analysis

The UniProt-GOA database^2^ was used for the ontological annotation of proteins. The KEGG database^3^ was used to investigate pathway enrichment. The InterPro database^4^ was used to analyze the enrichment of functional protein domains. The precise Fisher’s exact test was used to test the identified proteins against proteins in the database. FDR <0.05 in the enrichment analysis was statistically significant. NetSurfP_1.1^5^ was used to predict the secondary protein structure and surface accessibility.

#### Motif Discovery and Hierarchical Clustering Analysis

The MoMo analysis software, based on the motif-X algorithm, was used to analyze peptides sequences consisting of 10 amino acids upstream and downstream of all modification sites identified by proteome acetylation. When the number of peptides as a characteristic sequence exceeded 20 and the p-value of the statistical test was less than 0.000001, we considered the form of a characteristic sequence as a specific motif of modified peptides. Next, we used the “heatmap” function in the r-package “gplots” to display a location-specific heatmap. The hierarchical clustering method was then used to perform Gene Ontology (GO), KEGG, and structural domain analysis for the Q1-Q4 groups and the results were displayed as heat maps. GPS-PAIL 2.0^6^ software was then used to make simple predictions for seven acetyltransferases of the differentially acetylated proteins we identified.

#### Protein–Protein Interaction Network Analysis

First, the differential proteins identified between groups according to a multiple difference of 1.3 were analyzed by the STRING protein interaction database (V.11.0)^7^ to obtain a differential protein interaction network. Next, we utilized the STRING database to visualize the visual network between different proteins. MCODE and CytoHubba modules in Cytoscape software^8^ were then used to identify highly interconnected clusters and hub genes in the network.

## Results

### Identification and Analysis of the Global and Acetylated Proteome in TKPT Cells

**FIGURE 1:**
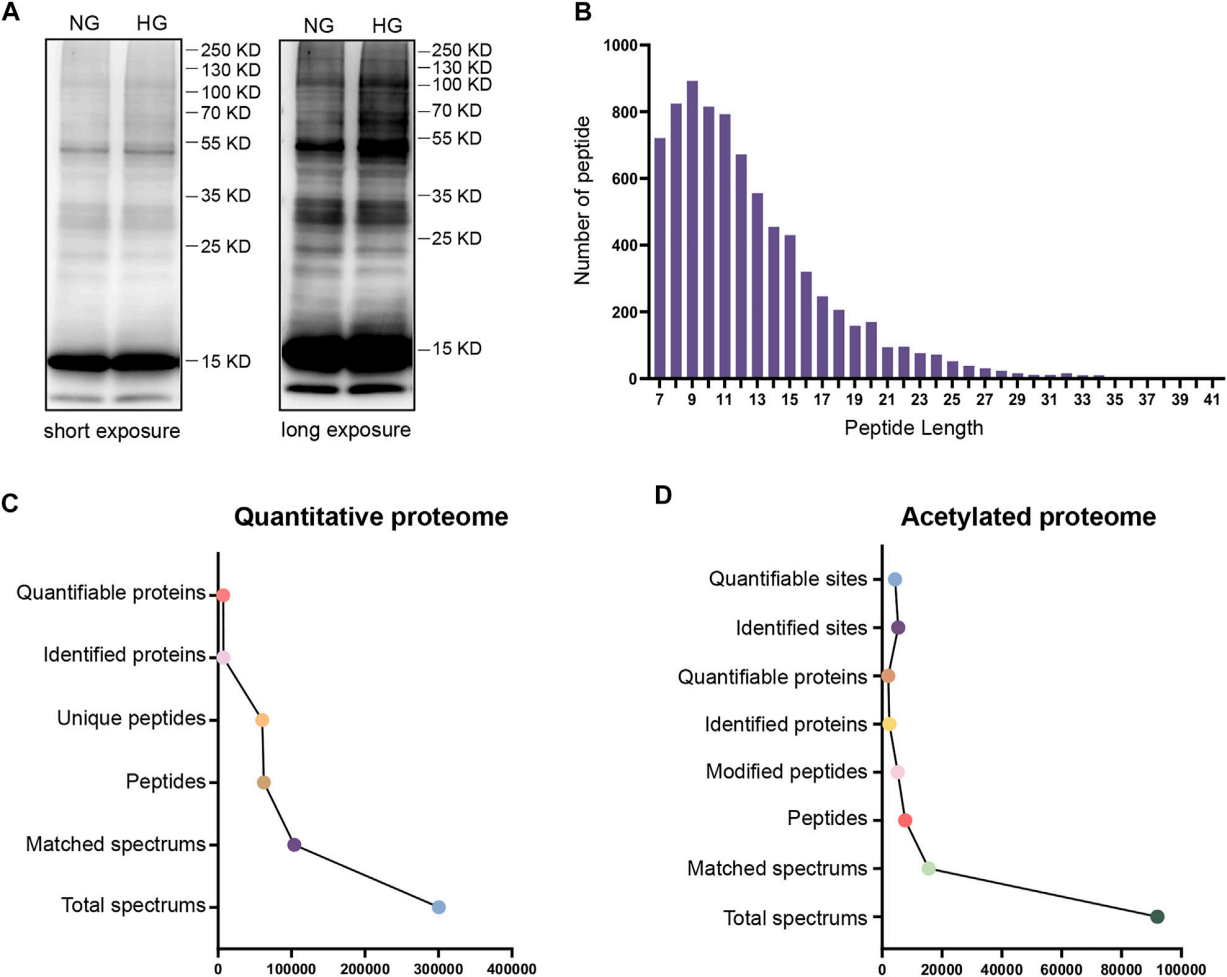
Analysis of the global proteome and acetylated proteome. **(A)** Western blotting of proteins in TKPT cells with anti-lysine acetylation antibodies. The two lanes represent NG (normal glucose: 5.6 mM) and HG (high glucose: 40 mM) conditions. Short exposure and long exposure, respectively. **(B)** The distribution of acetylated proteins based on the number of acetylation sites. **(C)** The global proteome was searched and analyzed in a MS/MS mass spectrometry database. **(D)** The acetylated proteome was searched and analyzed in a MS/MS mass spectrometry database.

To evaluate the degree of protein acetylation in TKPT cells under high glucose conditions, protein samples were isolated by SDS-PAGE and western blot analysis was conducted. Multiple bands of lysine-acetylated proteins, isolated from TKPT cells, showed strong and positive binding to an antibody against acetyl-lysine under high glucose conditions **(** Figure 1A). To further investigate the role of protein acetylation, the effect of high glucose on TKPT cells was determined by TMT labeling technology and liquid chromatography-tandem mass spectrometry. The distribution of mass error was near zero and most of them were less than 10 PPM, which means that the mass accuracy of the mass spectrum data fits the requirement **(** Supplementary Figure S1 **)**. In addition, the length of most peptides was between 7 and 20, agreeing with the properties of tryptic peptides **(** Figure 1B **)**. Principal component analysis (PCA) was also applied to verify the repeatability and the differences of the MS data **(** Supplementary Figure S2 **)**. We identified 6,924 proteins **(** Figure 1C, Supplementary Table S1 **)** and 4311 acetylation sites on 2012 acetylated proteins **(** Figure 1D **,** Supplementary Table S2 **)**. The acetylated proteins identified constituted about 29.06%(2012/6924) of the total proteins in mouse renal tubular epithelial cells, indicating that lysine acetylation is a common and key protein PTM in renal tubular epithelial cells.

### Differential Protein Identification and Subcellular Functional Localization

**FIGURE 2:**
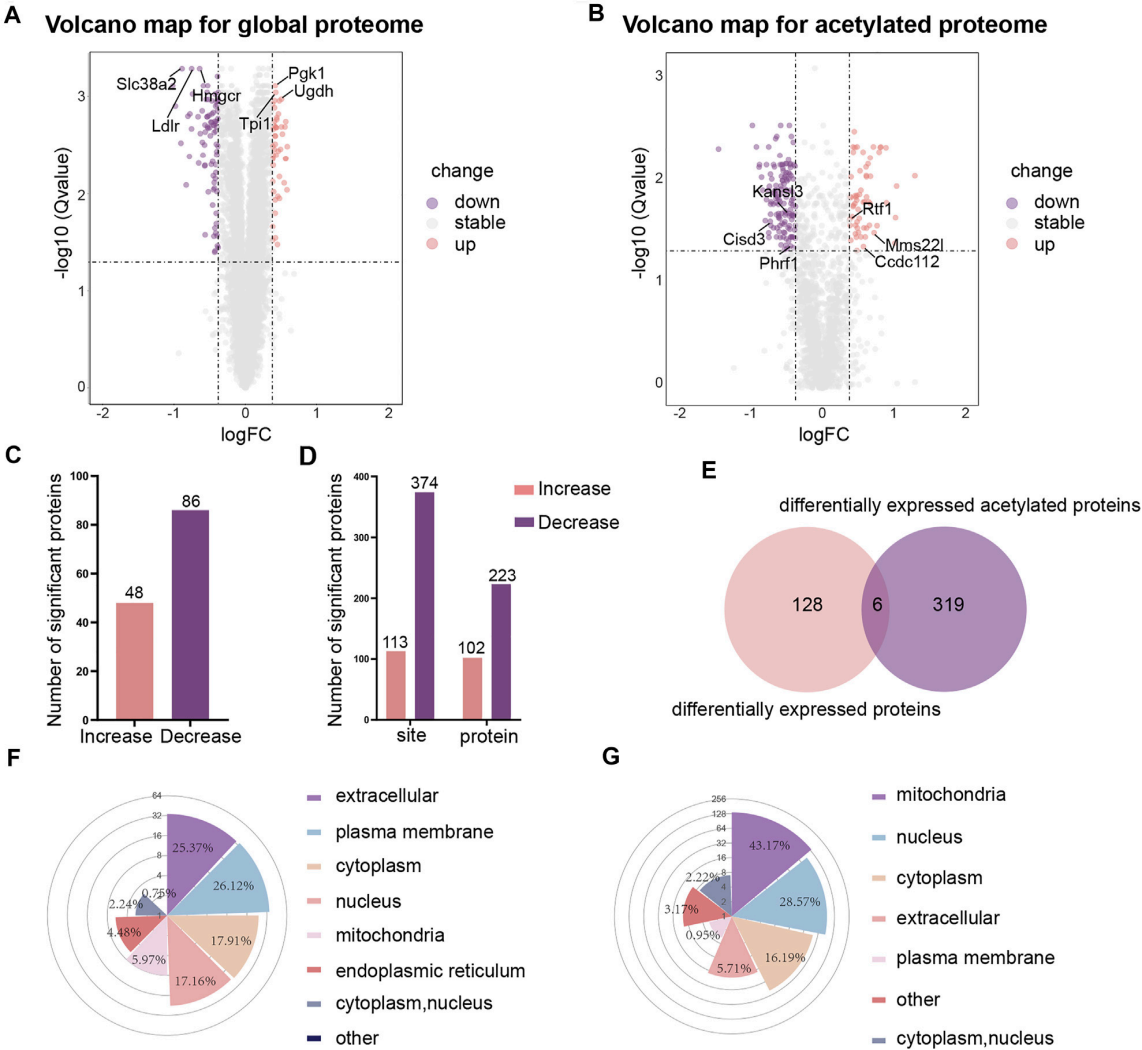
The differential analysis and subcellular localization of global proteome and acetylated proteome. **(A)** Volcano map of differentially expressed proteins in global proteome. **(B)** Volcano map of differentially expressed proteins in acetylated proteome. **(C)** Histograms showing differentially expressed proteins in global proteome. **(D)** Histograms showing differentially expressed acetylated proteins. **(E)** Venn diagrams showing differentially expressed proteins in global proteome and acetylated proteome. **(F)** Rose plots showing the subcellular localization of differentially expressed proteins in global proteome. **(G)** Rose plots showing the subcellular localization of differentially expressed acetylated proteins.

We were using a threshold of 1.3-fold change and an adjusted pvalue ≤0.05 to identify differentially expressed proteins. Volcano maps were used to visualize the differences in gene expression for global proteome **(** Figure 2A) and acetylated proteome **(** Figure 2B). Heat maps were also used to visualize the repeatability of differentially expressed proteins in three replicate samples of the experimental group and the control group (Supplementary Figure S3). Of the 6,924 proteins identified, 134 proteins were found to be differentially expressed. In comparison to the control group, 48 proteins were upregulated and 86 proteins were downregulated in the high-glucose group **(** Figure 2C **)**. When considering the acetylated proteins, we identified 113 sites with higher acetylation levels and 374 sites with lower acetylation levels in the high glucose group **(** Figure 2D **)**.

By comparing the differentially expressed proteins between the global proteome and the acetylated proteome (normalization of the acetylated proteins by the abundance of the respective protein have been performed in this work), we found that 128 proteins showed changes in expression levels but no change in the level of acetylation; 319 proteins had been acetylated but without any change in the total protein expression levels **(** Figure 2E **)**. Furthermore, we found that the acetylation levels of the three proteins Pgk1 (HG/NG = 1.338, HG: High-glucose, NG: Normal-glucose), Sub1 (HG/NG = 1.323), and Ctsz (HG/NG = 1.329) decreased while the total protein levels increased. The acetylation level of Crebrf (HG/NG = 0.739) increased while the total protein level decreased. The acetylation level and total protein level of Thbs1 (HG/NG = 0.689) were both decreased. Tcof1 (HG/NG = 0.676) was shown to feature sites with both increased and decreased levels of acetylation, although the total protein level decreased.

To better understand the distribution of the proteins in cells, we analyzed the subcellular localization of differentially expressed proteins for the global proteome and acetylated proteome. The results showed that for the global proteome, most of the differentially expressed proteins were distributed in the extracellular (25.37%), the plasma membrane (26.12%), the cytoplasm (17.91%), and the nucleus (17.16%) **(** Figure 2F **)**. The proteins with higher levels were mainly located in the cytoplasm (32.65%) and nucleus (30.61%), and the proteins with lower levels were primarily located in the plasma membrane (41.18%) and extracellular (28.24%). However, the subcellular localization analysis showed that the lysine-acetylated proteins were mainly located in the mitochondria (43.17%), nucleus (28.57%) and cytoplasm (16.19%) **(** Figure 2G **)**. The acetylated proteins with higher levels were predominantly located in the nucleus (64.21%), while the acetyled proteins with lower levels were primarily located in the mitochondria (58.64%).

### GO Enrichment Analysis of Differential Proteins

**FIGURE 3:**
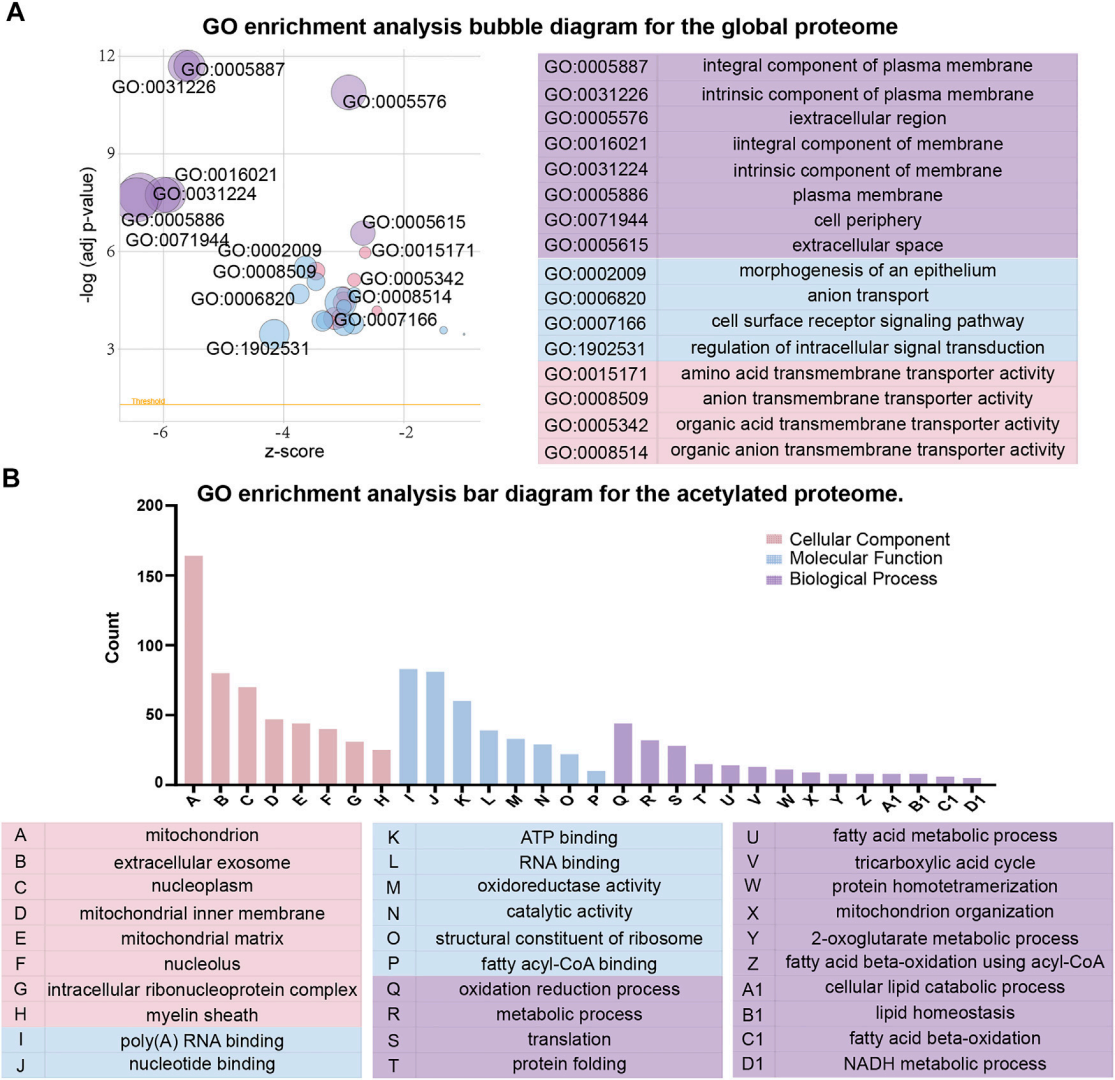
Gene ontology (GO) functional classification of differentially expressed proteins between the global proteome and acetylated proteome. **(A)** GO enrichment analysis bubble diagram for the global proteome. **(B)** GO enrichment analysis bar diagram for the acetylated proteome.

To better understand the effects of high glucose conditions on TKPT cells, we performed the GO enrichment analysis of the proteins that were differentially expressed in the global proteome **(** Figure 3A **)**. With regards to cellular components, the differentially expressed proteins were primarily enriched at the cell periphery, plasma membrane, and intrinsic component of the membrane. Of these, the differentially expressed proteins with higher levels were localized to the extracellular region and secretory granules and those with lower levels were bound to the membrane or localized to the cell periphery. With regards to molecular function, 17 proteins were involved in transmembrane transporter activity, 16 proteins were involved in ion transmembrane transporter activity, and 12 proteins were involved in anion transmembrane transporter activity. The molecular functions of differentially expressed proteins with lower levels were mainly associated with transmembrane transporter activity. However, the differentially expressed proteins with higher levels were primarily related to oxidoreductase activity and transcription factor binding. With regards to biological process, we identified that intracellular signal transduction, cell surface receptor signaling pathways, and ion transmembrane transport were significantly enriched. Similarly, differing from the proteins with lower levels, differentially expressed proteins with higher levels showed enrichment in exocytosis and the activation of granulocytes.

Next, with regards to cellular components, molecular function and biological process, we conducted the GO enrichment analysis on differentially expressed proteins in acetylated proteome (Figure 3B). The majority of acetylated proteins were associated with the mitochondria in the enrichment of cellular components. In terms of molecular function, catalytic activity and binding activity were significantly enriched. With regards to biological process, the most significant enrichment for acetylated proteins was related to metabolic processes, oxidation reduction process, and translation. However, the acetylated proteins with higher levels were primarily localized to nucleus, cytoplasm, and regulation of transcription in the GO enrichment analysis. We observed that most acetylated proteins with higher levels were localized to the regulation of genes, whereas the acetylated proteins with lower levels were localized to the catalytic activity of enzymes and metabolic processes.

This information helped us to better understand the relationship between changes in protein expression and associated functions, providing more clues to the pathogenesis of the disease.

### Domain Analysis and KEGG Enrichment Analysis of Differentially Expressed Proteins

**FIGURE 4:**
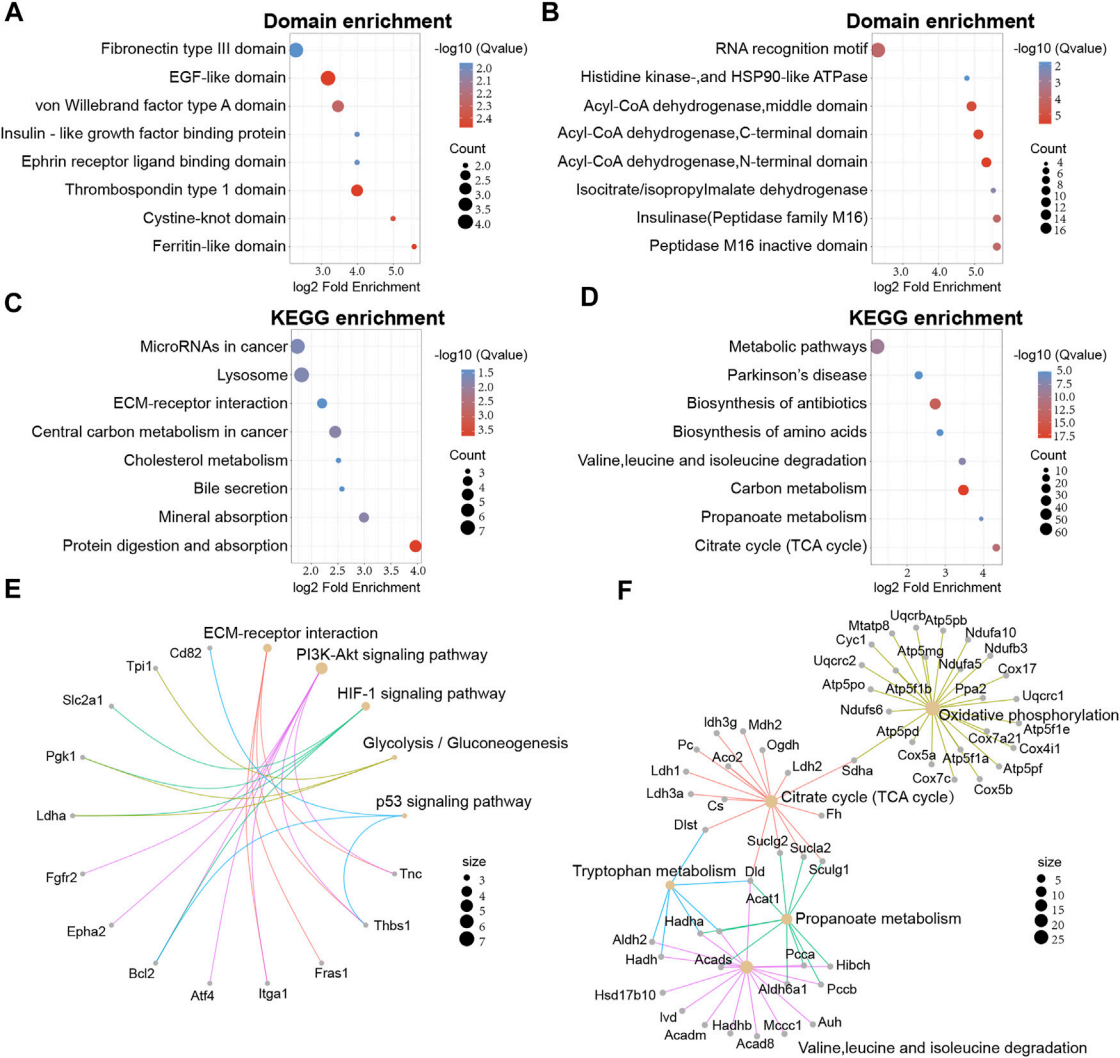
Protein domain and KEGG enrichment analysis of differentially expressed proteins in global proteome and acetylated proteome. **(A)** Domain enrichment analysis of the global proteome. **(B)** Domain enrichment of the acetylated proteome. **(C)** KEGG pathway enrichment analysis for the global proteome. **(D)** KEGG pathway enrichment analysis of the acetylated proteome. **(E)** Network map of specific KEGG pathways in the global proteome. **(F)** Network map of specific KEGG pathways in the acetylated proteome.

Next, we attempted to identify enrichment in specific domains of the differentially expressed proteins from the global proteome. Twelve protein domains were significantly enriched in the domain enrichment analysis, including von Willebrand factor type A domain, fibronectin type III domain, EGF-like domain, and thrombospondin type 1 domain (Figure 4A). With regards to the differentially expressed proteins that had been acetylated, 19 domains were significantly enriched, including RNA recognition motif, acyl-CoA dehydrogenase, and peptidase M16 inactive domain (Figure 4B). These results suggested that proteins with RNA recognition motif and acyl-CoA dehydrogenase domains may have a higher propensity for acetylation.

Secondly, KEGG enrichment analysis was performed to obtain a more comprehensive understanding on acetylated differentially expressed proteins of the global proteome. We found that 23 pathways were significantly enriched, including substance metabolism, microRNAs in cancer, and lysosomes. (Figure 4C). However, proteins with higher levels were mainly associated with glycolysis/gluconeogenesis and pentose and glucuronate interconversions. In addition, the acetylated differentially expressed proteins were also mapped to KEGG pathways. We identified 33 major pathways, including biosynthesis of antibiotics, TCA cycle, and the metabolism of some amino acids (Figure 4D).

To better understand the enrichment of important pathways and the interrelationships of the proteins within these pathways, we constructed a network diagram to visualize several pathways of interest. For the global proteome, we displayed five main pathways and we found that Thbs1, Bcl2 and Pgk1 were implicated in multiple pathways (Figure 4E). Meanwhile, five main pathways were displayed for the acetylated proteome and Dld, Hadha, and Sucla2 were implicated in multiple pathways (Figure 4F).

Next, to further understand the functional correlation of proteins, the differentially expressed proteins were further divided into four groups according to the extent of differential expression [Q1 (<0.667), Q2 (0.667–0.769), Q3 (1.3–1.5), and Q4 (>1.5)]. Then, GO enrichment analysis, KEGG enrichment analysis, and domain enrichment analysis were performed for each of these groups and the results were displayed as heat maps (Supplementary Figures S4, S5).

### Protein–Protein Interaction Network Analysis

**FIGURE 5:**
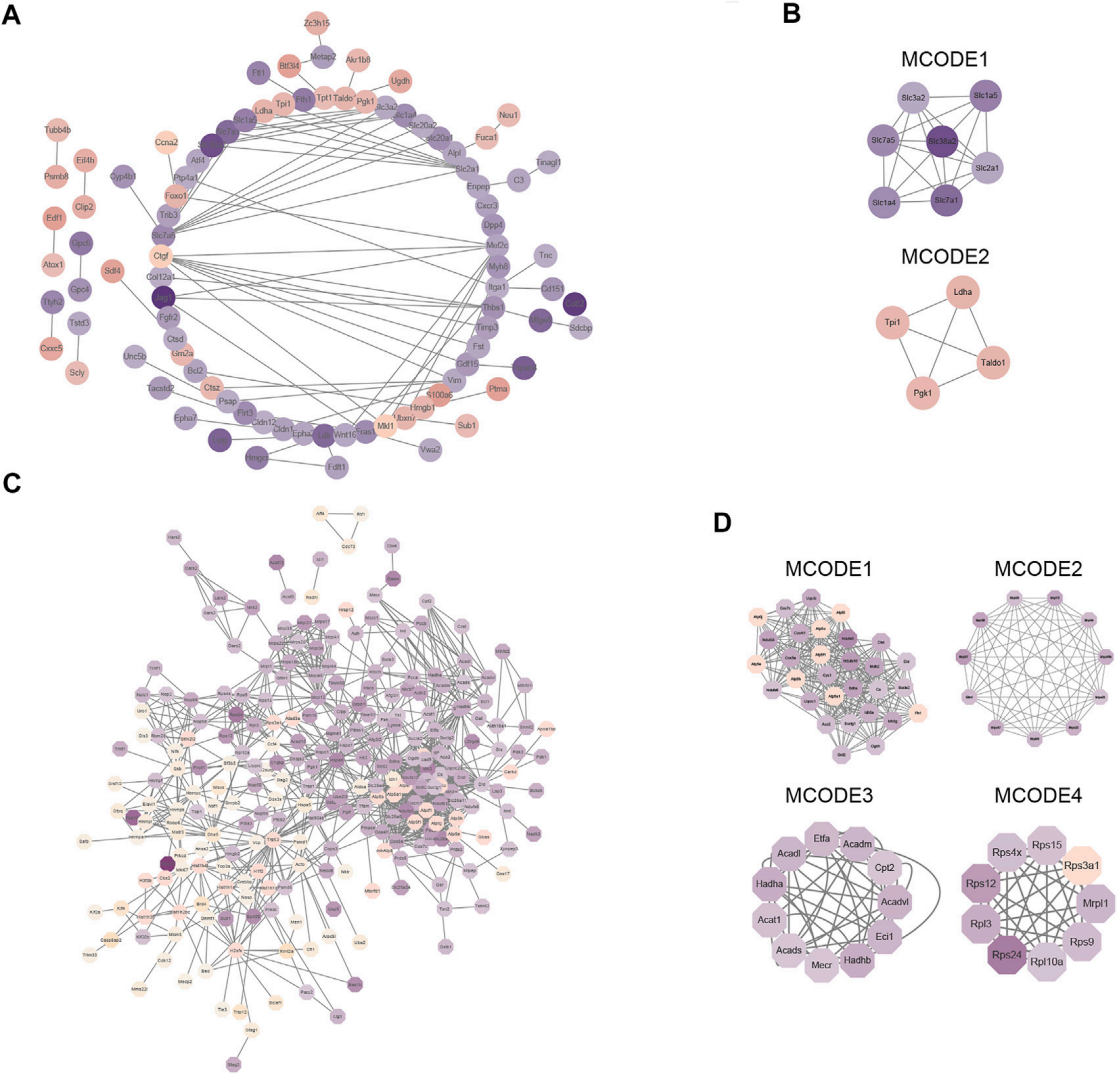
Protein–protein interaction (PPI) network analysis of differentially expressed proteins in global proteome and acetylated proteome. **(A)** STRING and Cytoscape software were used to create a PPI network for the global proteome. **(B)** Four modules of the global proteome. The four modules are based on the Molecular Complex Detection (MCODE) algorithm; “Degree Cutoff” was set to “4”. **(C)** STRING and Cytoscape software were used to create a PPI network for the acetylated proteome. **(D)** Four modules of the acetylated proteome. The four modules are based on the Molecular Complex Detection (MCODE) algorithm; “Degree Cutoff” was set to “4.”

To gain a better understanding of how the differentially expressed proteins relate to one another, we used the STRING database to construct a protein-protein interaction (PPI) network for the differentially expressed proteins of the global proteome and used Cystoscape to visualize the PPI network **(** Figure 5A **)**. Subsequently, we used MCODE plug-in and cytoHubba plug-in in Cytoscape software to identify several highly enriched MCODEs and hub genes. Two MCODEs were selected: MCODE 1 (MCODE score = 6.667) consisted of 7 nodes and 20 edges, MCODE 2 (MCODE score = 4) consisted of 4 nodes and 6 edges (Figure 5B **)**. Meanwhile, the top 10 hub proteins in the PPI network was selected by cytoHubba plug-in (Supplementary Table S3). In addition, we discovered that the proteins contained in MCODE 1 belonged to the family of solute carriers (SLC) and they are primarily responsible for the absorption and transportation of amino acids, nucleotides, glucose, and other substances on the cell membrane. Furthermore, the expression levels of these proteins were all reduced. Proteins in MCODE 2 were enzymes related to glycolysis and the expression levels of them were all increased.

Next, Cystoscape software was used to visualize the PPI network of differentially expressed proteins in the acetylated proteome (Figure 5C) and displayed the first four most compact MCODEs: MCODE 1 (MCODE score = 19.793) consisted of 30 nodes and 287 edges, MCODE 2 (MCODE score = 10.6) consisted of 11 nodes and 53 edges, MCODE 3 (MCODE score = 9) consisted of 11 nodes and 45 edges, and MCODE 4 (MCODE score = 8.5) consisted of nine nodes and 34 edges (Figure 5D). The top 10 hub proteins in the PPI network were also selected by cytoHubba plug-in **(** Supplementary Table S3 **)**. Meanwhile, we also found that proteins in MCODE 1 were principally associated with TCA cycle, those in MCODE 2 were mostly related to mitochondrial ribosomes, those in MCODE 2 were mostly related to lipid metabolism, and those in MCODE 4 were associated with ribosomal proteins.

### Distribution and Motif Analysis of Lysine Acetylation Sites

**FIGURE 6:**
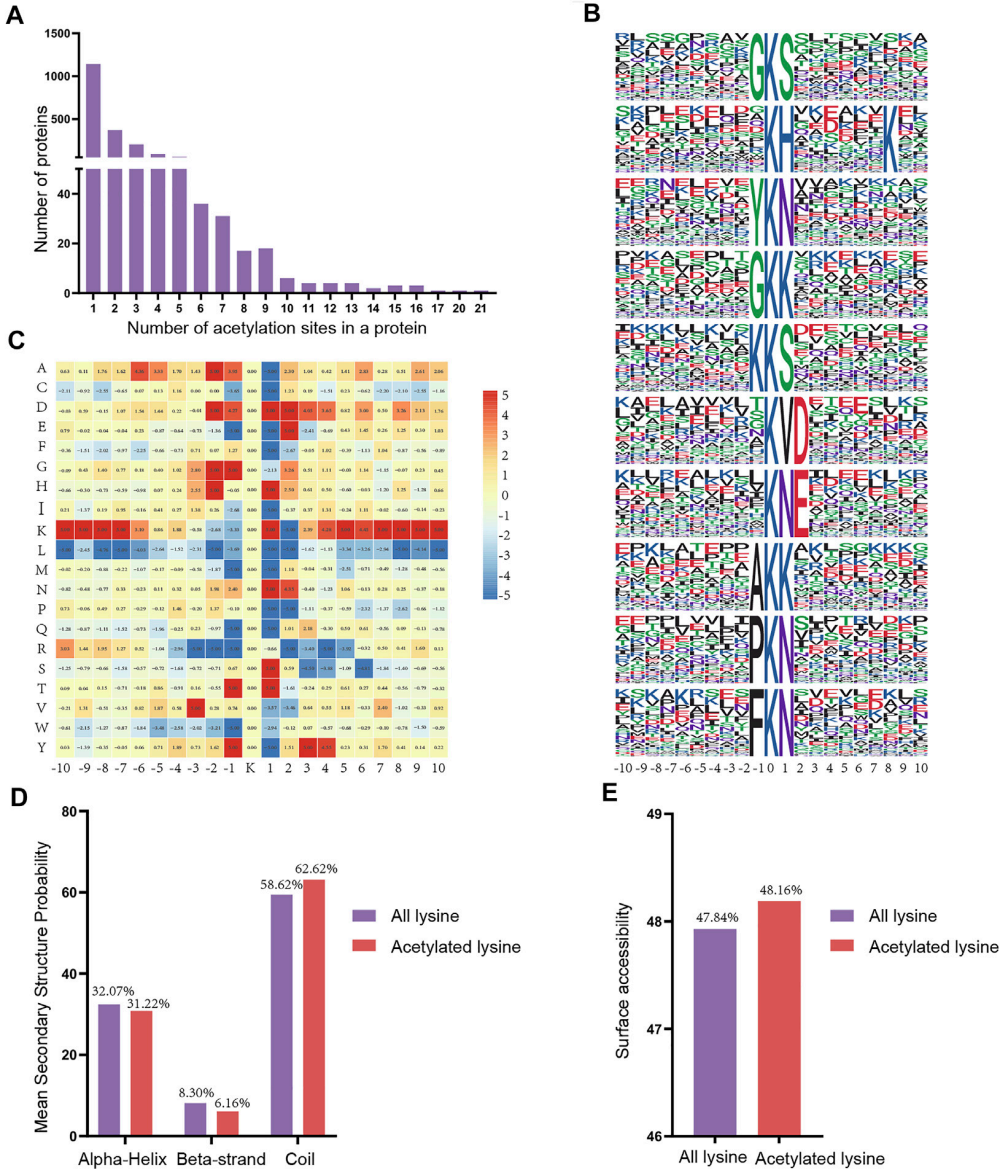
Properties of the acetylated peptides. **(A)** Distribution of acetylated proteins based on the numbers of acetylation sites. **(B)** Sequence probabilities of significantly enriched acetylation site motifs with 10 amino acids around the lysine acetylation site. **(C)** Heat map showing acetylation motif enrichment. **(D)** Secondary structure distribution of lysine acetylation sites. **(E)** Surface predictive accessibility of acetylation sites.

To assess the distribution of acetylation sites in proteins, we quantified the modified sites of acetylated proteins. We found that the proportion of proteins containing one acetylation site was 56.72% (1,143/2015), the proportion of proteins containing two acetylation sites was 18.56% (374/2015), and 10.27% (207/2015) of proteins contained three acetylation sites (Figure 6A). Notably, some of the acetylated proteins included over 10 acetylation sites, including Hspd1 with 17 acetylation sites, Hnrnpu with 20 acetylation sites, and Ncl with 21 acetylation sites.

Next, we used a motif-X program to investigate the amino acid motifs (10 amino acids upstream and downstream of the acetylation sites) surrounding the modification sites to further understand the characteristics of acetylation sites. A total of 38 conserved motifs were matched for the 4915 detected peptides and the first 10 motifs were displayed according to the motif score (Figure 6B, Supplementary Table S4). Enrichment of residues glycine (G), tyrosine (Y), lysine (K), alanine (A), proline (P) and phenylalanine (F) were found upstream of the acetylated lysine, whereas serine (S), histidine (H), lysine (K), asparagine (N), aspartic acid (D) and threonine (T) were found downstream of the acetylated sites. Depending upon the locations of these residues, the enrichment of most of the conservative residues (such as L, M, R and W) was significantly reduced in the vicinity of the Kac site. However, there is a strong preference for residues G, H, N and T in position ±1 or ±2 **(** Figure 6C **)**. Consequently, we hypothesized that proteins with thesee motifs are more susceptible to be recognized by lysine acetyltransferase.

Next, we compared the secondary structures around the acetylated lysines with those surrounding all lysines using the NetSurfP software **(** Figure 6D). The majority of acetylated lysines were located in disordered regions of proteins rather than in alpha-Helix (31.22%) and beta-strand (6.16%). Obviously, these results showed that the tendency for acetylation was not apparent. Analysis of the surface accessibility of acetylated lysine sites indicated that lysine acetylation might exert a slight influence on the surface properties of proteins (Figure 6E). Finally, we used GPS-PAIL 2.0 software to make simple predictions for the seven acetyltransferases of the acetylated differentially expressed proteins we identified to identify whether they were showed any significant regulatory relationships (Supplementary Table S5). The findings revealed that CREBBP and EP300 were the most abundant of the seven predicted acetyltransferases.

## Discussion

Diabetic nephropathy is considered to be one of the most common microvascular complications in diabetic patients. The pathogenesis of this disease is widely believed to be closely linked to damage to the structure and function of renal tubular epithelial cells (Chen et al., 2018; Han et al., 2019). Over the past few years, numerous studies have shown that the acetylation of proteins can exert regulatory effects on signaling pathways and gene expression (Sun et al., 2021). For instance, Lee et al. showed that Sirtuin1 (Sirt1) might facilitate the deacetylation of eNOS and change its enzyme activity (Fan et al., 2019). The regulation of the transcription factor FoxO3α by lysine deacetylase Sirt1 has been shown to alleviate oxidative stress and fibrosis in glomerular mesangium cells induced by high glucose conditions (Yang et al., 2019). Further, targeting the MRTF-A-associated epigenetic machinery might influence the tubulointerstitial fibrosis process of DN by interfering with the acetylation levels of histone (Xu et al., 2015). It follows then that the alteration of the acetylation levels of some specific proteins may affect the expression of downstream pathogenic genes. In other words, if more proteins with significantly changed acetylation levels can be found and their influence on disease activity can be verified by experiments, it will provide more insights to the mechanism and treatment strategies of DN. Due to the lack of related studies about acetylated proteome of renal tubular cells, it is urgent to explore the changes in global proteome and acetylated proteome in diabetic nephropathy.

In this paper, we determined the acetylated proteome of renal tubular cells with the identification of 2012 acetylated proteins and 4311 lysine acetylation sites. We also identified 102 acetylated proteins with higher levels and 223 acetylated proteins with lower levels in the high glucose group. Our results provides preliminary evidence for the widespread role of acetylation modification with respect to the damage subjected to high glucose in renal tubular epithelial cells. According to the subcellular location maps, the acetylated proteins with higher levels were predominantly localized in the nucleus and involved in the regulation of gene expression. In contrast, acetylated proteins with lower levels were primarily localized in the mitochondria and associated with various metabolic processes. Previous studies have shown that histones as the significant components of nucleosomes in the nucleus, the acetylation of them can affect the structure of chromatin and contribute to the regulation of gene expression (Li et al., 2016). It is worth mentioning that the change of acetylation levels of histones on the Connective tissue growth factor (CTGF) promoter region may influence the progression of DN (Shao et al., 2020). In addition, deacetylase Histone deacetylase 2 (HDAC2) is known to regulate the acetylation of other proteins and has also been shown to play a role in the apoptosis of renal tubular epithelial cells (Ma et al., 2016; Du et al., 2020). Our study also identified some proteins and enzymes associated with histones by screening from 325 acetylated differentially expressed proteins according to the related functional descriptions, including H1, H2, H3, H4 and histone acetyltransferases (Supplementary Table S6). These observations further suggest that the acetylation of histones may be involved in the pathogenesis of DN.

As introduced before, we identified six proteins (Pgk1, Sub1, Ctsz, Crebrf, Thbs1 and Tcof1) after comparing the differentially expressed proteins between global and acetylated proteomes. We provide the first evidence for the acetylation of the regulator of CREB3 recruiting factor (Crebrf) and transcription Sub1 in DN. Related studies have confirmed that PC4 (human ortholog gene of Sub1) plays a crucial role in maintaining a dynamic chromatin state (Das et al., 2010). By the way, proliferation, autophagy and apoptosis of cells may be influenced by regulating CREBRF/CREB3 pathway (Wu et al., 2019). Studies have also shown that Treacle Ribosome Biogenesis Factor 1 (TCOF1) can interact with deacetylase HDAC1 and may participate in the repair of DNA damage (Franek et al., 2016). In addition, we found that these proteins were also associated with glucose metabolism, the p53 signaling pathway, the TGF-beta signaling pathway, phagosomes, and other pathways (Wang et al., 2015; Qian et al., 2017; Sun et al., 2019; Frampton et al., 2020). Meanwhile, TGF-beta and P53 were thought to be involved in renal interstitial fibrosis and inflammation (Ma et al., 2019; Cappelli et al., 2020). Thus, we could speculate that the lysine acetylation of these proteins may plays a significant role in several important pathways and has potential value in influencing the progression of DN.

KEGG pathway enrichment analysis identified that the acetylated proteins identified in our study were localized in a variety of pathways, including TCA cycle, amino acid metabolism, and the HIF-1α pathway. With a wide range of target genes, hypoxia-inducible factor-1 alpha (HIF-1α) has been demonstrated to be involved in EMT under high glucose conditions in renal tubular epithelial cells (Ndibalema et al., 2020). It has been reported that the CREB Binding Protein (CREBBP, P300/CBP, HG/NG: 1.458 fold at K1584, 1.372 fold at K1807) is a common acetyltransferase that can bind to HIF-1α and initiates hypoxia (Wei et al., 2018). Meanwhile, the combination of CREBBP and HIF-1α can also act on the downstream enzymes, such as Pgk1 (HG/NG: 0.744 at Kac388, 0.669 at Kac30, 0.702 at Kac291), Aldolase A (Aldoa, HG/NG: 1.496 at Kac230), and Hexokinase 2 (Hk2, HG/NG: 0.65 at Kac418) to promote anaerobic reactions and influence TGF-β-dependent fibrosis processes (Ye et al., 2018; Yin et al., 2019). Interestingly enough, the increased acetylation level of PGK1 at K323 may promote its enzyme activity and exacerbate the EMT process in ovarian cancer cells (Hu et al., 2017; Gou et al., 2021). Furthermore, as one of the downstream enzymes of HIF-1α, PDK-1 (HG/NG: 0.688AT Kac212) is known to be associated with apoptosis and the TCA cycle (Saurus et al., 2015; Heinemann-Yerushalmi et al., 2021). In brief, these findings does not rule out of the possibility that lysine acetylation may play a regulatory role in the HIF-1 α pathway and may also affect apoptosis and EMT.

**FIGURE 7:**
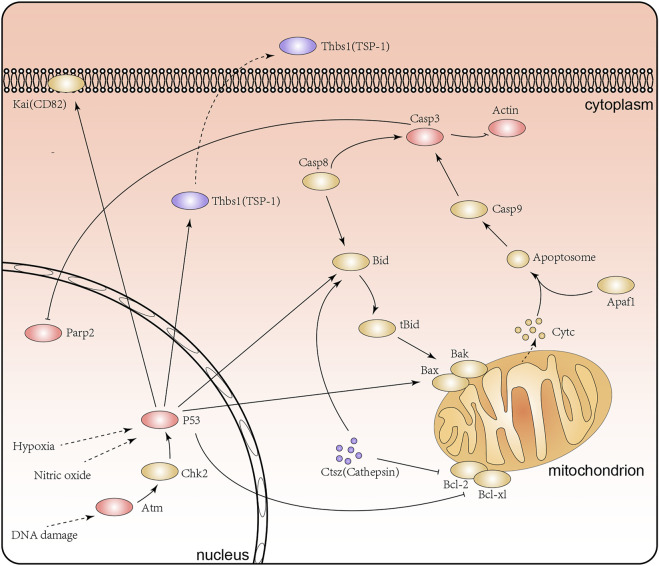
The apoptosis signaling pathway and P53 signaling pathway in the renal tubular epithelial cells under high glucose conditions. Purple represents proteins with lower levels of acetylation, red represents proteins with higher levels of acetylation, and yellow represents proteins with no significant difference in acetylation levels.

Notably, our research surprisingly found that a range of proteins involved in apoptosis signaling pathway and p53 signaling pathway, including Tp53 (HG/NG: 1.756 at Kac367, 1.448 at Kac317), Atm (HG/NG: 1.412 at Kac384), Casp3 (HG/NG: 1.473 at Kac25), Thbs1 (TSP1, HG/NG: 0.621 at Kac587), Parp2 (HG/NG:1.304 at Kac36, 0.768 at Kac51), Actb (HG/NG: 1.531 at Kac50), and Ctsz (HG/NG: 0.715 at Kac66) (Figure 7). Existing studies have shown that the inhibition of apoptosis and the p53 signaling pathway in renal tubular epithelial cells may improve renal lesions in DN (Patel et al., 2019). The acetylation of P53 at K317 is known to negatively regulate its apoptotic activity, while the increased acetylation level at K36/37 of PARP-2 has been shown to reduce DNA binding and enzyme activitiies (Chao et al., 2006; Haenni et al., 2008). Moreover, Atm not only acts on p53 but also plays a role in repairing damaged DNA by increasing the level of acetylation at K3106 (Wang et al., 2019; Hwang et al., 2020). Further, the inhibition of Casp3 activity has been shown to reduce PARP cleavage and therefore influence apoptosis (Sairanen et al., 2009). However, there are few reports for the influence of lysine acetylation of Casp3 and Ctsz proteins in DN. Collectively, we discovered the acetylation of Atm (K384), Casp3 (K25), Parp2 (K51), Ctsz (K66) and Actb (K50) under high glucose for the first time. Combining previous research and our acetylated proteome analysis, we inferred that lysine acetylation of these proteins might affect the structure and function of renal tubular epithelial cells by the apoptosis signaling pathway and the p53 signaling pathway.

Because lysine acetylation is mostly regulated by acetyltransferases and deacetylases, we used GPS-PAIL 2.0 software to make simple predictions for the seven acetyltransferases of the differentially acetylated proteins we quantified. Previous studies have shown that the down-regulation of acetyltransferase KAT3B induces apoptosis in renal carcinoma and the variations in the expression levels of MYSTI/MOF/KAT8 may affect the cell cycle and the EGFR signaling pathway (Cocco et al., 2018; Dong et al., 2019). Additionally, our prediction results identified certain acetyltransferases with high scores, such as KAT8 which can act on Parp2 (K36/K51 site) and Casp3 (K25 site). In the meantime, we found that the acetylation levels of Crebbp, Kat8, and Kat7 were significantly increased under high glucose conditions while their total expression levels remained the same. To our knowledge, these findings have not been reported previously. Nevertheless, these results provide more information for understanding the regulatory relationships between acetyltransferase and deacetylase on some important proteins. According to previous reports, the regulation of the enzyme activities can affect the course of the disease by affecting protein acetylation levels (Vancura et al., 2018; Chung et al., 2019; Nakamura, 2021). Overall, it is necessary to carry out in-depth research on the relationships of the acetylation levels in responding to the enzymes for the important proteins.

Although the findings from this study revealed for the first time that many proteins are acetylated under high glucose conditions, there are still some limitations to our study that need to be considered. For example, it is possible that some critical proteins may have been omitted due to mass spectrometry technology and the threshold settings used for differential analysis. Furthermore, our results require additional experimental testing.

## Conclusion

In brief, in this study, we attempted to build a comprehensive proteome and post-translational modification profiles of renal tubular epithelial cells. A total of 6,924 proteins were confidently identified, with 4311 lysine acetylation sites listed from 2012 acetylated proteins. Most importantly, we compared the expression levels of acetylated proteins of the control group and the high-glucose group and found some acetylated differentially expressed proteins associated with apoptosis, p53 signaling pathway and other signaling pathways. Although these results call for further experimental comparative studies, our research provides rich resources for studying the pathogenesis of DN, and these differentially expressed proteins may be employed as new targets for treatment and prevention in diabetic nephropathy.

## Data Availability

The mass spectrometry proteomics data have been deposited to the ProteomeXchange Consortium via the PRIDE partner repository with the dataset identifier PXD029651.

## Footnotes

1
https://www.atcc.org/

2
http://www.ebi.ac.uk/GOA/

3
https://www.genome.jp/kegg/

4
http://www.ebi.ac.uk/interpro/

5
www.cbs.dtu.dk/services/NetSurfP-1.1/

6
http://pail.biocuckoo.org/

7
http://string-db.org

8
http://www.cytoscape.org/

## Supplementary Material

Supplementary Figure S1
**(A)** Mass error distribution of all peptides in the global proteome. **(B)** Mass error distribution for all peptides in the acetylated proteome.

Supplementary Figure S2
**(A)** PCA analysis for the global proteome. **(B)** PCA analysis for the acetylated proteome.

Supplementary Figure S3
**(A)** Heat map for differentially expressed proteins in the global proteome. **(B)** Heat map for differentially expressed proteins in the acetylated proteome.

Supplementary Figure S4 GO, KEGG and domain enrichment analysis for differentially expressed proteins in the global proteome. **(A)** GO (biological process) enrichment clustering analysis for the four groups (Q1-Q4). **(B)** GO (cellular component) enrichment clustering analysis for the four groups (Q1-Q4). **(C)** GO (molecular function) enrichment cluster analysis for the four groups (Q1-Q4). **(D)** KEGG pathway enrichment cluster analysis for the four groups (Q1-Q4). **(E)** Domain enrichment clustering analysis for the four groups (Q1-Q4).

Supplementary Figure S5 GO, KEGG and domain enrichment analysis for differentially expressed proteins in the acetylated proteome. **(A)** GO (biological process) enrichment clustering analysis for the four groups (Q1-Q4). **(B)** GO (cellular component) enrichment clustering analysis for the four groups (Q1-Q4). **(C)** GO (molecular function) enrichment cluster analysis for the four groups (Q1-Q4). **(D)** KEGG pathway enrichment cluster analysis for the four groups (Q1-Q4). **(E)** Domain enrichment clustering analysis for the four groups (Q1-Q4).

Supplementary Table S1 Global proteome.

Supplementary Table S2 Acetylated proteome.

Supplementary Table S3 Top ten hub proteins.

Supplementary Table S4 Motif‐x.xlsx.

Supplementary Table S5 The prediction of Acetyltransferase.xls.

Supplementary Table S6 Proteins associated with histones.

## Nomenclature

**Resource Identification Initiative:** 1) TKPTS cell line, ATCC Cat# CRL-3361, RRID:CVCL_UJ13; 2) anti acetyllysine antibody, PTM BIO Cat# PTM-105, RRID:AB_2877698; 3) MaxQuant, RRID:SCR_014485; 4) UniProt database, Universal Protein Resource, RRID:SCR_002380; 5) KEGG, RRID:SCR_012773; 6) InterPro, RRID:SCR_006695; 7) NetSurfP, RRID:SCR_018781; 8) motif-X, Motif Extractor, RRID:SCR_008628; 9) GPS-PAIL, RRID:SCR_021671; 10) STRING, RRID:SCR_005223.
