# Supplementary material for: Lysine Acetylation in the Proteome of Renal Tubular Epithelial Cells in Diabetic Nephropathy
Source: Front Genet. 2021 Nov 25;12:767135. doi: 10.3389/fgene.2021.767135 (PMC8657754; doi:10.3389/fgene.2021.767135)
Supplement: Supplementary file 1 [file Table6.doc]

**Table S6.** Proteins associated with histones that were subjected to altered levels of acetylation.

| **Gene name** | **Description** | **Position** | **HG/NG Ratio** | **Qvalue** |
| --- | --- | --- | --- | --- |
| H1-0  H1-1  H1-1  H1-3  H1-3  H2ax  H2bc4  H2bc4  H2bc4  H3-3a  H3-3a  H3c2  H4c1  H4c1  H4c1  Kmt2a  Crebbp | Histone H1.0  Histone H1.1  Histone H1.1  Histone H1.3  Histone H1.3  Histone H2AX  Histone H2B  Histone H2B  Histone H2B  Histone H3.3  Histone H3.3  Histone H3.2  Histone H4  Histone H4  Histone H4  N-methyltransferase  Acetyltransferase | K55  K22  K66  K65  K64  K134  K6  K44  K109  K15  K28  K28  K78  K80  K92  K1519  K1807 | 0.582  1.377  0.731  0.716  0.762  1.485  1.548  0.760  0.754  1.329  1.748  1.765  0.732  0.584  0.673  2.326  1.372 | 0.00898836  0.00749166  0.00831145  0.01546106  0.03398071  0.00579285  0.02014546  0.04457831  0.02538708  0.01478946  0.00521271  0.00515176  0.00813561  0.00743239  0.00765035  0.00985098  0.02214964 |

Gene name, Description, Position, HG/NG Ratio and Qvalue were obtained by the Mass spectrometry.
