# Supplementary figures and images for: Lysine Acetylation in the Proteome of Renal Tubular Epithelial Cells in Diabetic Nephropathy
Source: Front Genet. 2021 Nov 25;12:767135. doi: 10.3389/fgene.2021.767135 (PMC8657754; doi:10.3389/fgene.2021.767135)

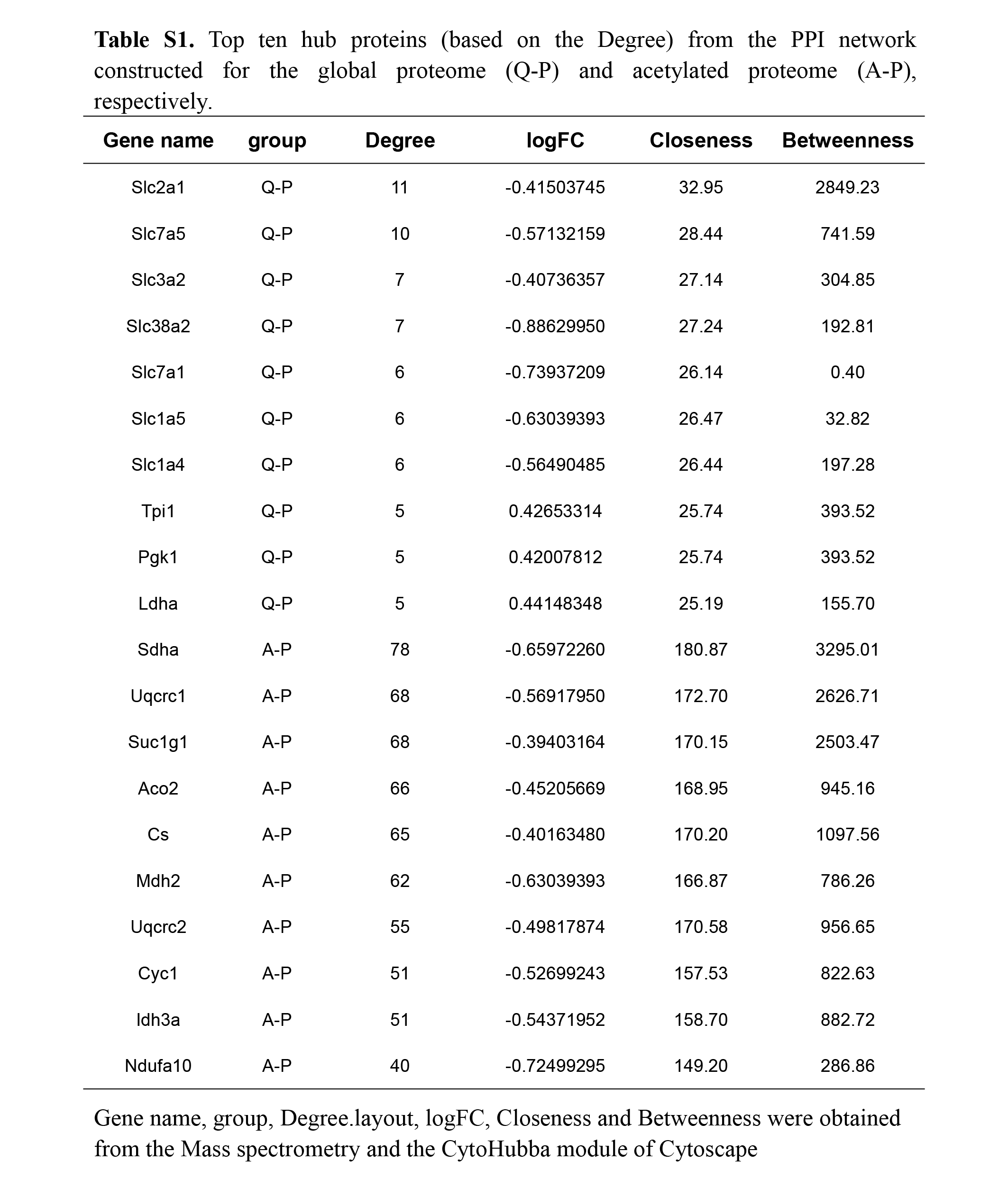

Supplement: Supplementary file 4 [file Image6.TIF]

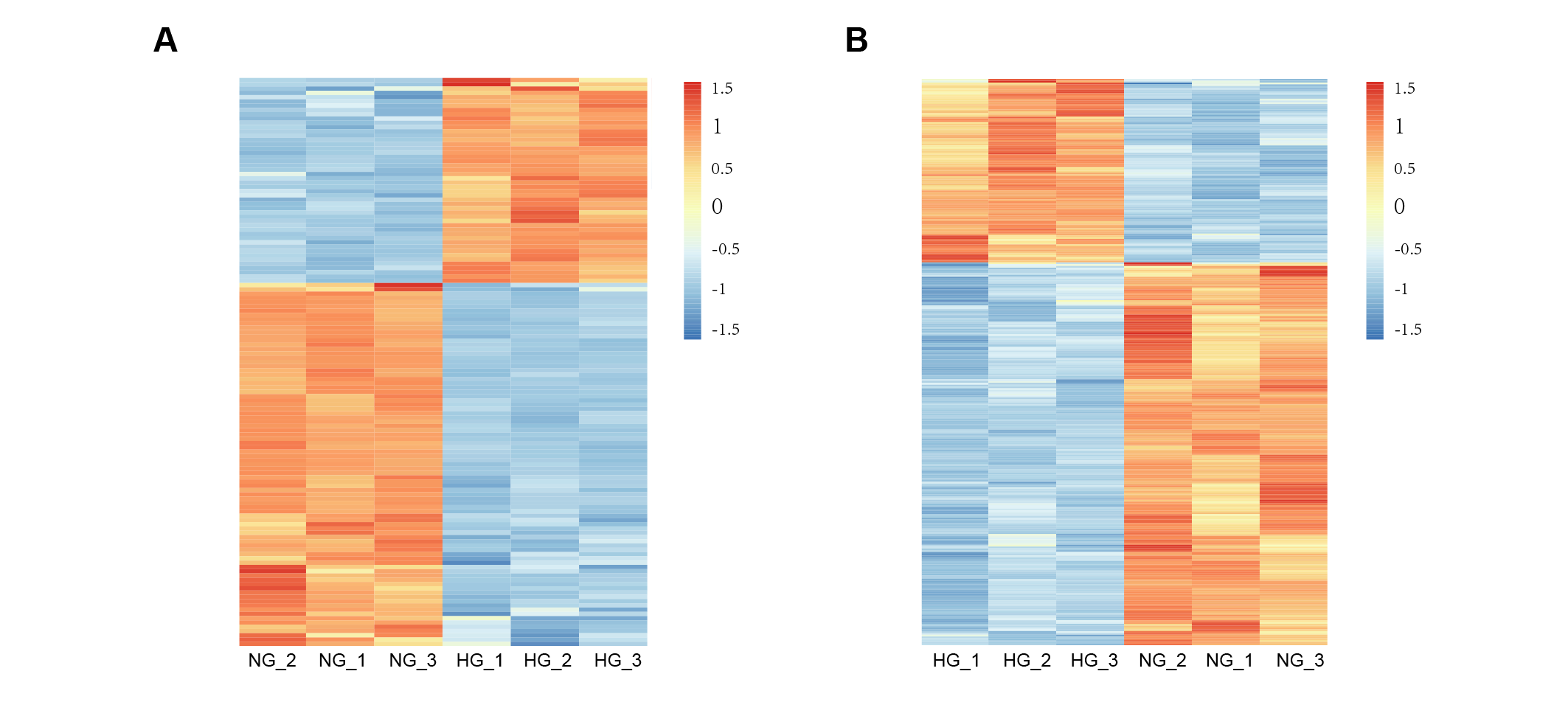

Supplement: Supplementary file 5 [file Image3.TIF]

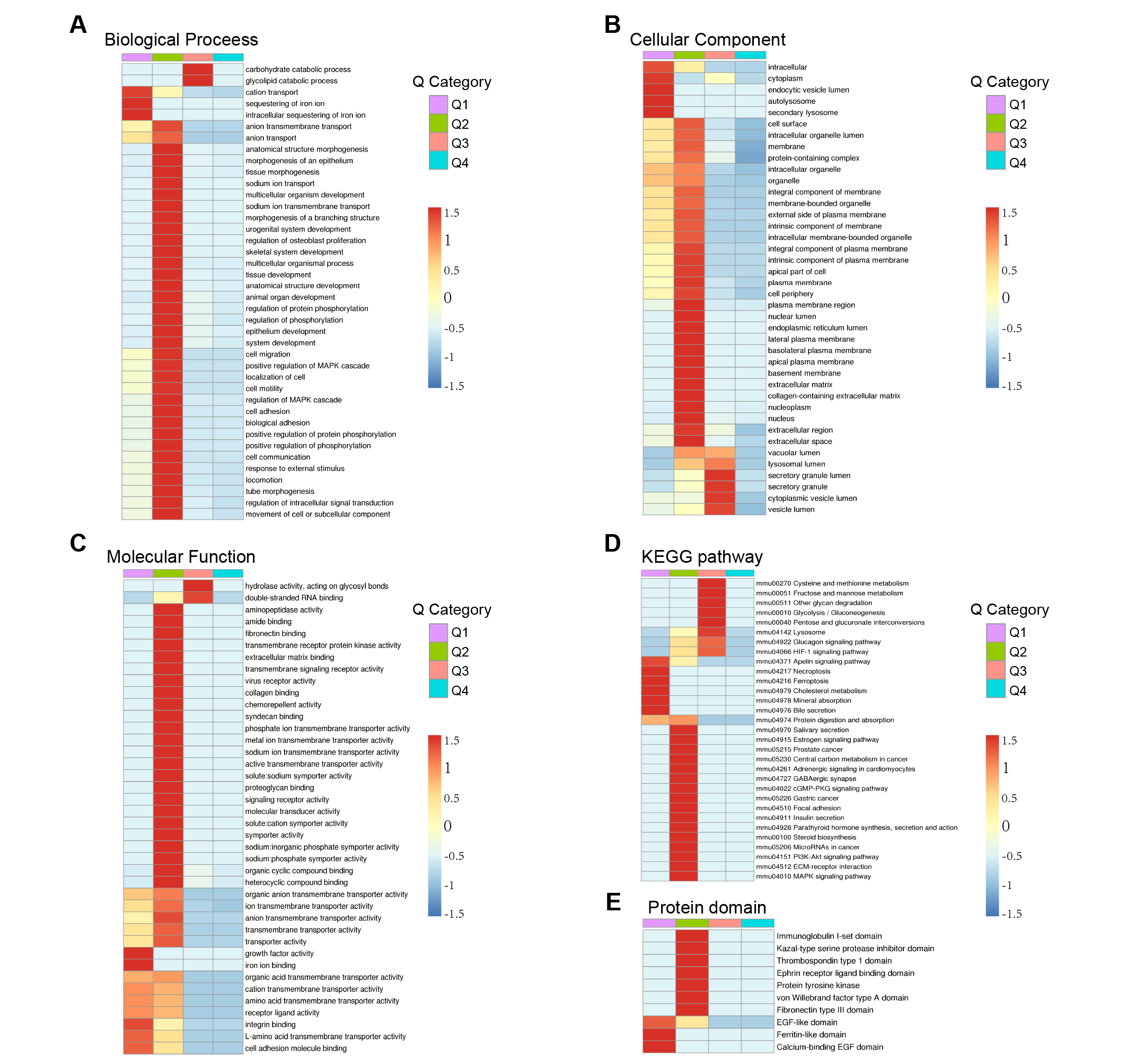

Supplement: Supplementary file 6 [file Image4.TIF]

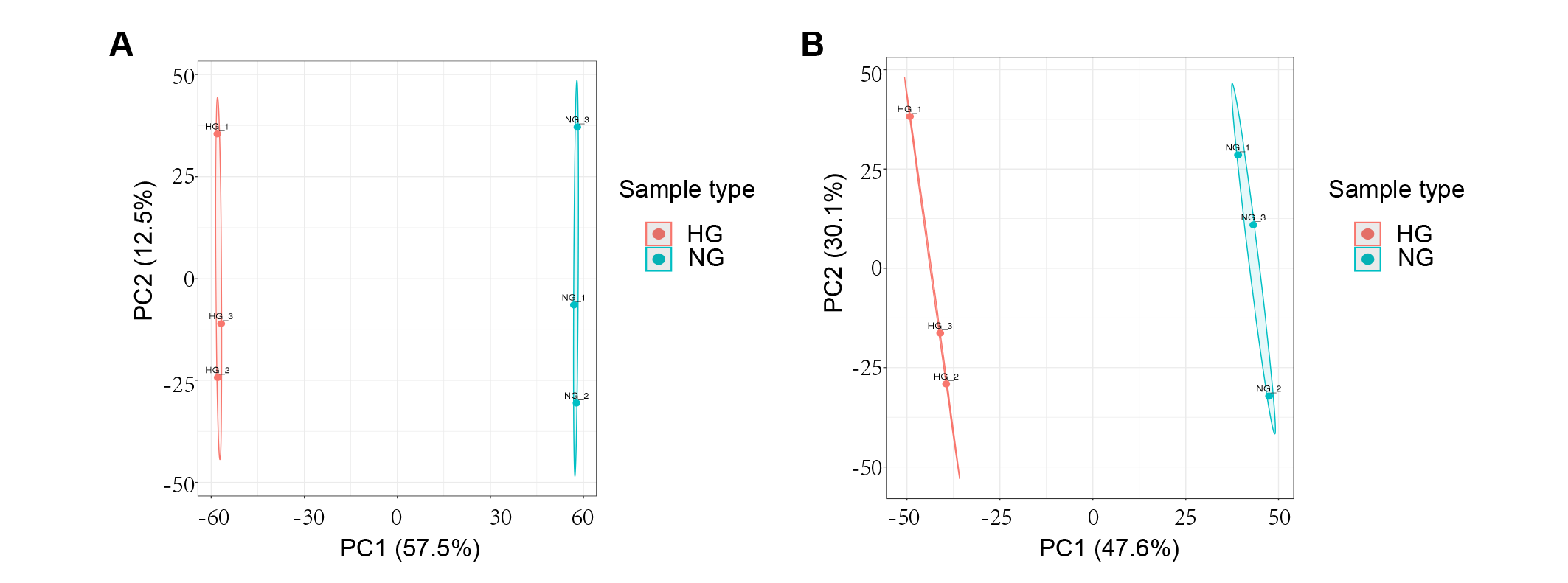

Supplement: Supplementary file 9 [file Image2.TIF]

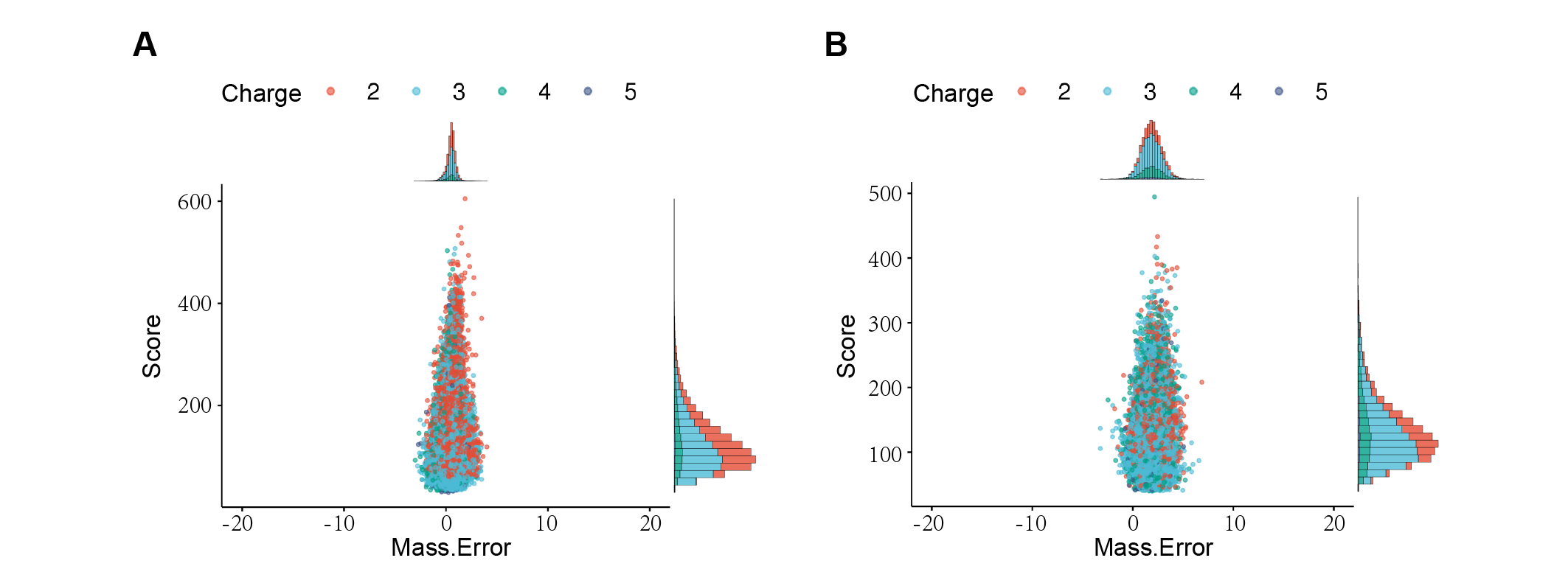

Supplement: Supplementary file 10 [file Image1.TIF]

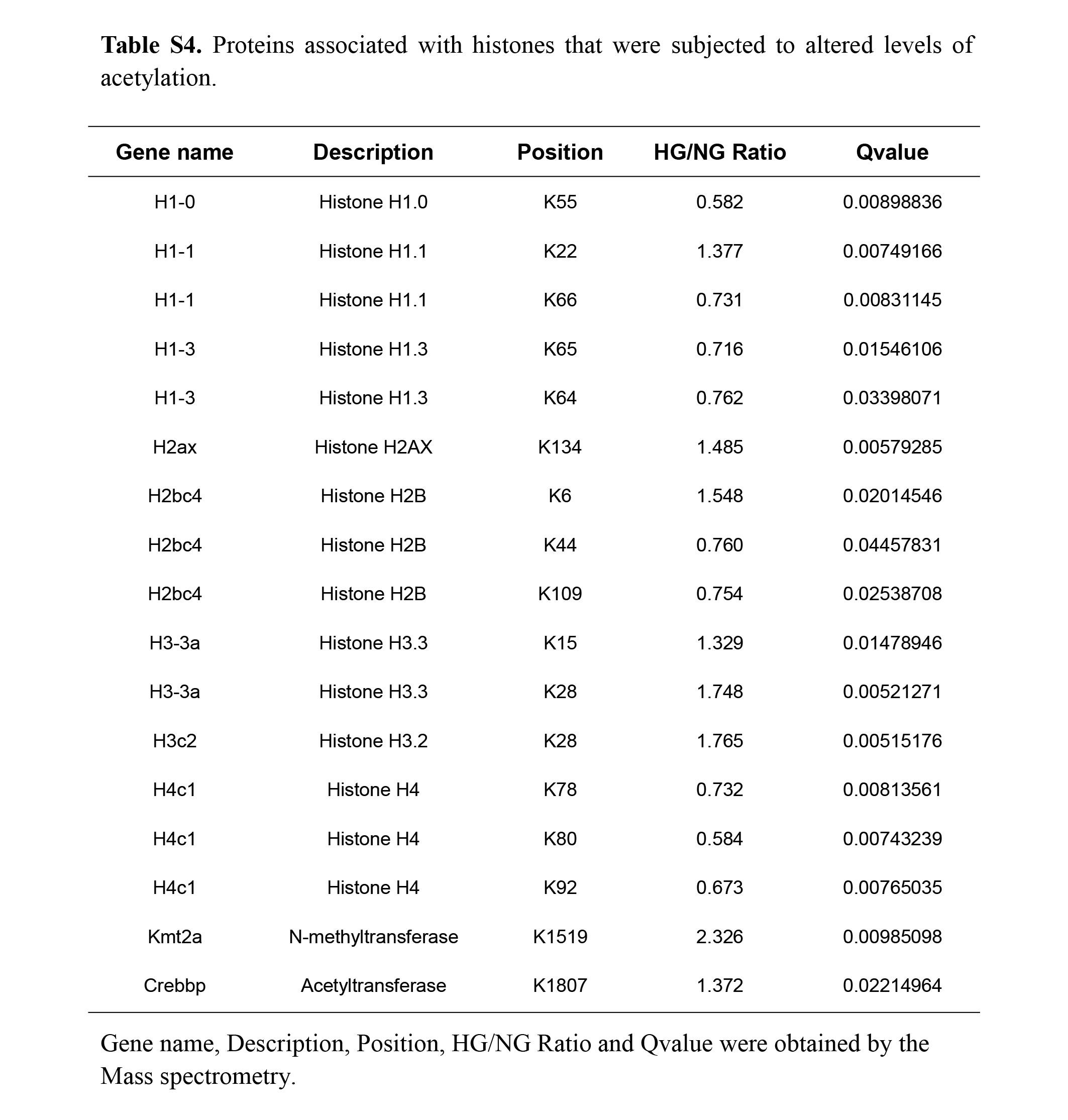

Supplement: Supplementary file 11 [file Image7.TIF]

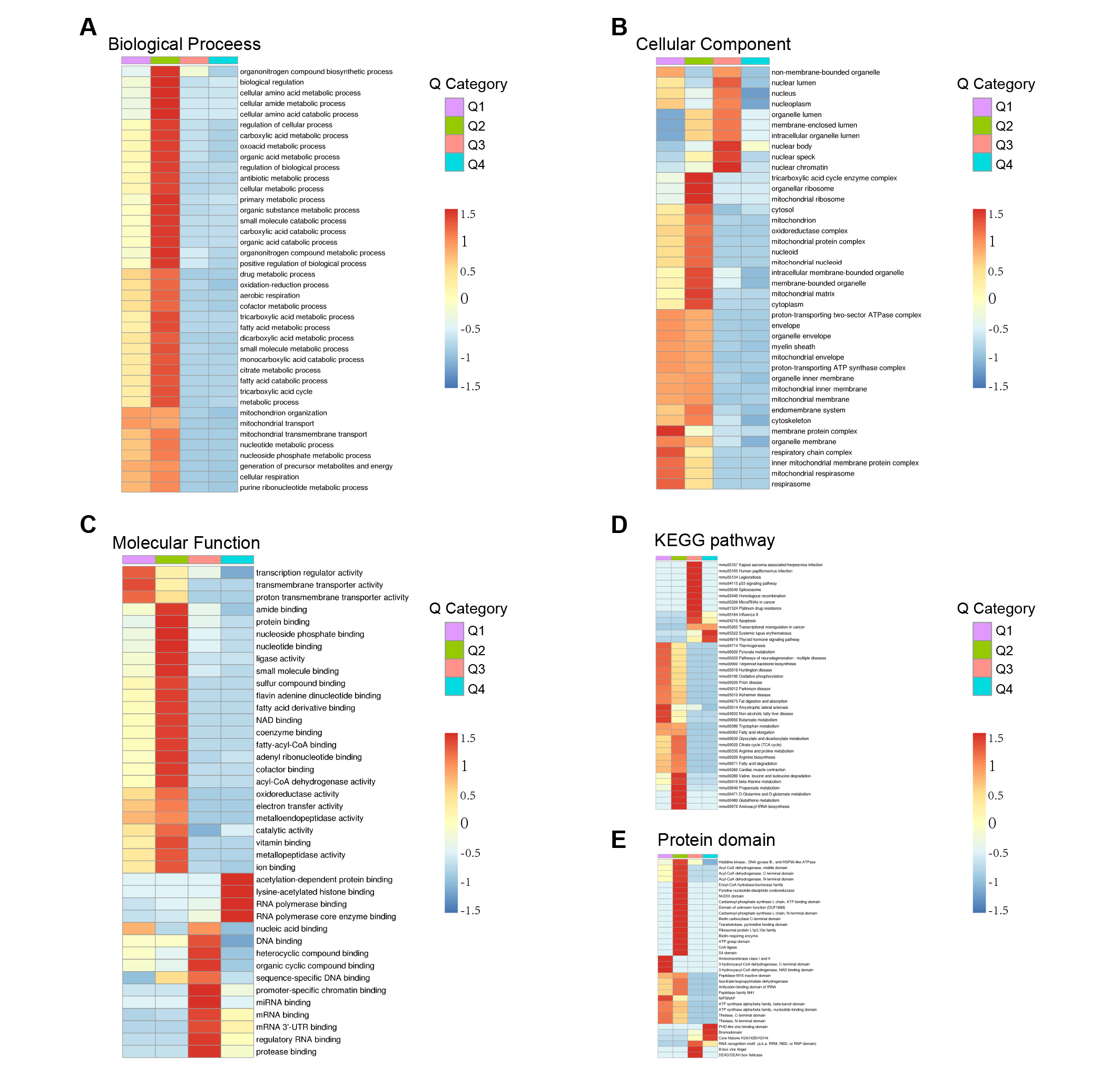

Supplement: Supplementary file 13 [file Image5.TIF]
