# Supplementary material for: Lysine Acetylation in the Proteome of Renal Tubular Epithelial Cells in Diabetic Nephropathy
Source: Front Genet. 2021 Nov 25;12:767135. doi: 10.3389/fgene.2021.767135 (PMC8657754; doi:10.3389/fgene.2021.767135)
Supplement: Supplementary file 7 [file Table3.doc]

**Table S3.** Top ten hub proteins (based on the Degree) from the PPI network constructed for the global proteome (Q-P) and acetylated proteome (A-P), respectively.

| **Gene name** | **group** | **Degree** | **logFC** | **Closeness** | **Betweenness** |
| --- | --- | --- | --- | --- | --- |
| Slc2a1  Slc7a5  Slc3a2  Slc38a2  Slc7a1  Slc1a5  Slc1a4  Tpi1  Pgk1  Ldha  Sdha  Uqcrc1  Suc1g1  Aco2  Cs  Mdh2  Uqcrc2  Cyc1  Idh3a  Ndufa10 | Q-P  Q-P  Q-P  Q-P  Q-P  Q-P  Q-P  Q-P  Q-P  Q-P  A-P  A-P  A-P  A-P  A-P  A-P  A-P  A-P  A-P  A-P | 11  10  7  7  6  6  6  5  5  5  78  68  68  66  65  62  55  51  51  40 | -0.41503745  -0.57132159  -0.40736357  -0.88629950  -0.73937209  -0.63039393  -0.56490485  0.42653314  0.42007812  0.44148348  -0.65972260  -0.56917950  -0.39403164  -0.45205669  -0.40163480  -0.63039393  -0.49817874  -0.52699243  -0.54371952  -0.72499295 | 32.95  28.44  27.14  27.24  26.14  26.47  26.44  25.74  25.74  25.19  180.87  172.70  170.15  168.95  170.20  166.87  170.58  157.53  158.70  149.20 | 2849.23  741.59  304.85  192.81  0.40  32.82  197.28  393.52  393.52  155.70  3295.01  2626.71  2503.47  945.16  1097.56  786.26  956.65  822.63  882.72  286.86 |

Gene name, group, Degree.layout, logFC, Closeness and Betweenness were obtained from the Mass spectrometry and the CytoHubba module of Cytoscape
